# Supplementary material for: Empirical methods for controlling false positives and estimating confidence in ChIP-Seq peaks
Source: BMC Bioinformatics. 2008 Dec 5;9:523. doi: 10.1186/1471-2105-9-523 (PMC2628906; doi:10.1186/1471-2105-9-523)
Supplement: Additional File 1 — A variety of html documents from the USeq web site detailing the available applications, their best usage, output file type descriptions, command line menus, etc. [file 1471-2105-9-523-S1.zip › USeqUserGuides/JavaDoc.zip!/JavaDoc/util/bio/digest/Enzyme.html]

Enzyme


|  |  |  |
| --- | --- | --- |
| **Overview** \| **Package** \| **Class** \| **Use** \| **Tree** \| **Deprecated** \| **Index** \| **Help** | |  |
| PREV CLASS   **NEXT CLASS** | **FRAMES**    **NO FRAMES**     **All Classes** |  |
| SUMMARY: NESTED \| FIELD \| CONSTR \| METHOD | DETAIL: FIELD \| CONSTR \| METHOD |  |


---


## util.bio.digest Class Enzyme

```
java.lang.Object
  util.bio.digest.Enzyme
```

**All Implemented Interfaces:**: java.lang.Comparable

---

``` public class Enzyme extends java.lang.Object implements java.lang.Comparable ```

Restriction enzyme information.

---

**Constructor Summary**

`Enzyme(java.lang.String aName,
java.lang.String aRecogSeq,
java.lang.String aMethSens,
java.lang.String aAvail,
java.lang.String aIsoz)`


| **Method Summary** | |
| --- | --- |
| `int` | `compareTo(java.lang.Object obj)` |
| `java.lang.String` | `getAvail()` |
| `int` | `getCutBottom()` |
| `int` | `getCutTop()` |
| `java.lang.String` | `getIso()` |
| `int` | `getLength()` |
| `java.lang.String` | `getMethSens()` |
| `java.lang.String` | `getName()` |
| `int` | `getNumberOfCuts()` |
| `char` | `getOverHangs()` |
| `java.lang.String` | `getRecogSeq()` |
| `java.lang.String` | `getRegEx()` |
| `java.lang.String` | `getStrippedRecogSeq()` |
| `int[]` | `getTopCuts()` |
| `void` | `printEnz()` |
| `void` | `setTopCuts(int[] topCuts)` |

**Methods inherited from class java.lang.Object**

`equals, getClass, hashCode, notify, notifyAll, toString, wait, wait, wait`

**Constructor Detail**

### Enzyme

```
public Enzyme(java.lang.String aName,
              java.lang.String aRecogSeq,
              java.lang.String aMethSens,
              java.lang.String aAvail,
              java.lang.String aIsoz)
```


**Method Detail**

### getName

```
public java.lang.String getName()
```

---


### getRecogSeq

```
public java.lang.String getRecogSeq()
```

---


### getMethSens

```
public java.lang.String getMethSens()
```

---


### getAvail

```
public java.lang.String getAvail()
```

---


### getIso

```
public java.lang.String getIso()
```

---


### getCutTop

```
public int getCutTop()
```

---


### getCutBottom

```
public int getCutBottom()
```

---


### getLength

```
public int getLength()
```

---


### getRegEx

```
public java.lang.String getRegEx()
```

---


### getOverHangs

```
public char getOverHangs()
```

---


### printEnz

```
public void printEnz()
```

---


### getTopCuts

```
public int[] getTopCuts()
```

---


### setTopCuts

```
public void setTopCuts(int[] topCuts)
```

---


### compareTo

```
public int compareTo(java.lang.Object obj)
```

:   **Specified by:**: `compareTo` in interface `java.lang.Comparable`

---


### getNumberOfCuts

```
public int getNumberOfCuts()
```

---


### getStrippedRecogSeq

```
public java.lang.String getStrippedRecogSeq()
```


---


|  |  |  |
| --- | --- | --- |
| **Overview** \| **Package** \| **Class** \| **Use** \| **Tree** \| **Deprecated** \| **Index** \| **Help** | |  |
| PREV CLASS   **NEXT CLASS** | **FRAMES**    **NO FRAMES**     **All Classes** |  |
| SUMMARY: NESTED \| FIELD \| CONSTR \| METHOD | DETAIL: FIELD \| CONSTR \| METHOD |  |


---
